# Vesicular Glutamate Release from Feeder-FreehiPSC-Derived Neurons

**DOI:** 10.3390/ijms231810545

**Published:** 2022-09-11

**Authors:** Simona Baldassari, Chiara Cervetto, Sarah Amato, Floriana Fruscione, Ganna Balagura, Simone Pelassa, Ilaria Musante, Michele Iacomino, Monica Traverso, Anna Corradi, Paolo Scudieri, Guido Maura, Manuela Marcoli, Federico Zara

**Affiliations:** 1 Unit of Medical Genetics, IRCCS Istituto Giannina Gaslini, Via G. Gaslini 5, 16147 Genova, Italy; 2 Department of Pharmacy (DIFAR), Section of Pharmacology and Toxicology, University of Genoa, Viale Cembrano 4, 16148 Genova, Italy; 3 Interuniversity Center for the Promotion of the 3Rs Principles in Teaching and Research (Centro 3R), 56100 Pisa, Italy; 4 Department of Neurosciences, Rehabilitation, Ophthalmology, Genetics, Maternal and Child Health (DINOGMI), University of Genoa, Largo Paolo Daneo 3, 16132 Genova, Italy; 5 Paediatric Neurology and Neuromuscular Disorders Unit, IRCCS Istituto Giannina Gaslini, Via G. Gaslini 5, 16147 Genova, Italy; 6 Department of Experimental Medicine, University of Genoa, Viale Benedetto XV 3, 16132 Genova, Italy; 7 IRCCS Ospedale Policlinico San Martino Largo Rosanna Benzi 10, 16132 Genova, Italy; 8 Center of Excellence for Biomedical Research, Viale Benedetto XV, 16132 Genova, Italy

**Keywords:** stem cells, human-induced pluripotent stem cells (hiPSC), human neurons, diseases modelling, neurotransmitter release

## Abstract

Human-induced pluripotent stem cells (hiPSCs) represent one of the main and powerful tools for the in vitro modeling of neurological diseases. Standard hiPSC-based protocols make use of animal-derived feeder systems to better support the neuronal differentiation process. Despite their efficiency, such protocols may not be appropriate to dissect neuronal specific properties or to avoid interspecies contaminations, hindering their future translation into clinical and drug discovery approaches. In this work, we focused on the optimization of a reproducible protocol in feeder-free conditions able to generate functional glutamatergic neurons. This protocol is based on a generation of neuroprecursor cells differentiated into human neurons with the administration in the culture medium of specific neurotrophins in a Geltrex-coated substrate. We confirmed the efficiency of this protocol through molecular analysis (upregulation of neuronal markers and neurotransmitter receptors assessed by gene expression profiling and expression of the neuronal markers at the protein level), morphological analysis, and immunfluorescence detection of pre-synaptic and post-synaptic markers at synaptic boutons. The hiPSC-derived neurons acquired Ca^2+^-dependent glutamate release properties as a hallmark of neuronal maturation. In conclusion, our study describes a new methodological approach to achieve feeder-free neuronal differentiation from hiPSC and adds a new tool for functional characterization of hiPSC-derived neurons.

## 1. Introduction

Human induced Pluripotent Stem Cells (hiPSCs) are considered a unique and well-characterized resource to develop advanced cell models of human origin, such as neuroprecursors (NPCs) and neurons, in addition to glial and also retinal cells, from easily accessible somatic cells to investigate human developmental and disease mechanisms. The hiPSC-derived cell models are also suitable for testing personalized pharmacological and cellular therapeutic approaches and neurotoxicity screenings. Moreover, human-derived in vitro models provide an alternative experimental approach to animal-derived cells to avoid interspecies differences, reducing uncertainties in results translation and improving the prediction of human toxicity [1,2,3].

These hiPSCs represented a breakthrough in experimental neuroscience. Improved protocols allowed differentiation of hiPSCs into mature neuronal and glial cells for modeling key neurodevelopmental or neurodegenerative processes and testing developmentally adverse neurotoxicity [4,5,6] in different neurological conditions such as autism spectrum disorder, schizophrenia, Alzheimer’s disease, Parkinson’s Disease, amyotrophic lateral sclerosis, and fragile X syndrome [7,8,9,10,11,12,13,14].

A central issue when reprogramming human neurons from iPSCs is the identification of robust assays and biomarkers to monitor the complex processes underlying cell specification, differentiation, and functional maturation and to uncover disease-related physiopathological mechanisms. Morphological analysis of hiPSC-derived neurons, for instance, provides important readouts on neuronal development. At the first stages of the differentiation process, alteration of neurite outgrowth and impairment of synapse formation, leading to modification of electrical activity, emerged as common physiological hallmarks for neurodevelopmental disorders [11,12,13]. For instance, hiPSC-derived NPCs from autism spectrum disorder patients showed an increase in proliferation and a reduction of excitatory neural progenitor cell markers. Furthermore, the differentiation of NPCs in mature human neurons displayed a deficit of GABAergic components and a decrease inexcitatory pre-synapses [15,16]. It has been shown that in hiPSC-derived neurons, the expression of neuronal markers precedes the appearance of functional properties, such as action potential firing and synapse formation [17,18,19]. Therefore, the expression of proteins such as Neuronal Nuclear protein (NeuN)/Fox-3 or Microtubule-associated protein 2 (MAP2) is considered a hallmark of neuronal maturity [20]. Analysis of the transcriptome of single hiPSC-derived GABAergic and glutamatergic neurons revealed more than 300 transcripts upregulated in mature neurons that represent potential cell-specific maturity markers [21]. Electrophysiology represents the golden standard to assess the functional maturation of human neurons [21]. The establishment of 70–90 mV resting potential and the ability to fire evoked action potential bursts are critical functional markers of neuronal maturation [18,19]. Additionally, Ca^2+^-imaging was used to study human neuron maturation [22,23] and to monitor neuron excitability, the midbrain dopamine (DA) neuron pacemaker activity [24] as well as the functioning of neurotransmitter receptors [25,26]. The acquired expression of functional ionotropic glutamatergic receptors as N-methyl-D-aspartate (NMDA) receptors is also considered a marker of neuronal maturation [25,27], as required for high-level organization of the network activity [28,29].

The acquisition of a mature neuronal phenotype implicates the ability to sense and communicate through chemical signaling, i.e., through neurotransmitters. Although exocytotic vesicular Ca^2+^-dependent release of neurotransmitters is a key neuron-specific endpoint, the ability of hiPSC-derived neurons to release neurotransmitters has been rarely evaluated. Furthermore, studies exploring the intracellular signaling pathways in hiPSC-derived neuronal cultures are still lacking [17].

To this aim, we devised a neuronal differentiation protocol starting from iPSCs and NPCs generated from neural rosettes, radially organized columnar epithelial cells with a central lumen mimicking the structure of the developing neural tube [30] to test its ability to generate hiPSC-derived neurons. In most approaches, differentiating neurons are co-cultured with rat or mouse astrocytes or with conditioned medium to achieve full maturation [31]. The novel feature of our protocol is the technique of obtaining mature hiPSC-derived neurons: we focused on developing a standardized protocol to obtain a homogeneous and stable population of human neurons, exploitable as a cellular model to characterize neurogenic pathologies and for future pharmacological approaches. More in-depth, NPCs were plated in a feeder-free state on a Geltrex coating and small molecules were added into the medium to generate a population of cells exhibiting the features typical of mature neurons in 30–45 days of differentiation in vitro (DIV). We showed that these human neurons express neural-related markers and acquire a neuronal phenotype. Finally, we assessed the ability of hiPSC-derived neurons to release glutamate upon depolarization and its Ca^2+^-dependency, providing a novel insight into the functional properties of hiPSC-derived neurons.

## 2. Results

### 2.1. Generation and Characterization of iPSCs and Neuronal Differentiation

**Figure 1:**
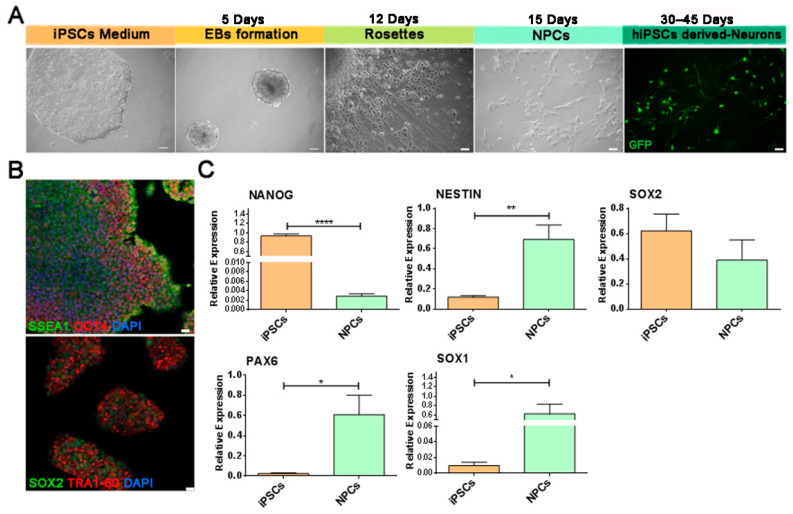
Differentiation from human-induced pluripotent stem cells (hiPSCs) to neuronal cells. (**A**) Schematic diagram showing the differentiation protocol: from left to right, hiPSCs, Embyoid Bodies (EBs), Neuronal Rosettes, sun-shaped structure with rays from the center (Rosettes), Neuroprecursors (NPCs) and hiPSC-derived Neurons. Scale bar 10 µm. (**B**) Representative images of immunofluorescence (IF) of embryonic stem cell surface markers: Stage specific embryonic antigen-1 (SSEA1; green) and Octamer-binding transcription factor 4 (OCT4; red) (**top** image), and SRY-Box Transcription Factor 2 (SOX2; green) and T cell receptor alpha locus 1-60 (TRA-1-60; red) (**bottom** image). Cells were stained with nuclear marker 4′,6-diamidino-2-phenylindole, DAPI (blue). Scale bar 10 µm. (**C**) RT-qPCR shows upregulation of Sox1 and Pax6 in NPCs, as compared to hiPSCs. Nestin and Sox2 involved in pluripotency and neuronal differentiation are expressed in NPCs and hiPSCs. Bar graphs show the mean values ± SEM of relative expression, at least *n* = 3 replicate for each group. * *p* < 0.05, ** *p* < 0.01, **** *p* < 0.0001, by one way ANOVA, Bonferroni’s multiple comparisons.

The generation of functional active human neurons from iPSCs relies on a differentiation protocol that includes different stages from the initial in vitro generation of NPCs based on culture media enriched by basic fibroblast growth factor (bFGF), retinoic acid (RA), Brain-Derived Neurotrophic Factor (BDNF), and Glial cell-derived neurotrophic factor (GDNF). An outline of the protocol is described in Figure 1 [32,33,34]. The hiPSCs were initially generated from fibroblasts derived from a healthy donor by transducing four transcription factors, *Octamer-binding transcription factor 4* (*Oct4)*, *Kruppel Like Factor 4* (*Klf4)*, *SRY-Box Transcription Factor 2* (*Sox2)*, and *C-myc* with Sendai 2.0 virus [35]. Characterization of hiPSC cell lines showed a strong expression of the typical pluripotent stem cell markers, Oct4, Stage specific embryonic antigen-1 (Ssea1), Sox2, and T cell receptor alpha locus (Tra)1-60 (Figure 1B) by immunofluorescence (IF) analysis and the absence of genomic rearrangements in iPSC clones by Array-CGH analysis (Supplementary Figure S1). To exploit neural differentiation, hiPSCs were dissociated to single cell suspension for the generation of 3D aggregates, known as embryoid bodies (EBs) (Figure 1A). The neuronal lineage induction was elicited through the generation of 2D radially organized cell structures, called neuronal rosettes [36,37,38] (Figure 1A). In our protocol, neuronal rosettes were generated by plating the EBs on Geltrex-coated plates and, with the addition of the Neuronal Induction medium supplemented with the SMADi, this promotes neuroectodermal induction by blocking TGF-β and Bone Morphogenetic Protein-dependent SMAD (small mother against decapentaplegic) signaling. NPC populations were then isolated from the neural rosettes and expanded (Figure 1A). Transcriptional profile of NPCs showed downregulation of the pluripotency marker Homeobox protein Nanog and upregulation of the neuronal precursor genes, compared to the hiPSC line. Nestin and Sox2, genes of self-renewal, were significantly expressed in both cell lines; neural ectodermal markers Pax6 and Sox1 were upregulated in NPCs compared to iPSCs (Figure 1C). As outlined in Figure 1A, NPCs were induced to differentiate with specific growth factors: BDNF, GDNF, and RA. After 30–45 DIV in feeder-free conditions, the cells showed a morphology consistent with mature neurons (Figure 1A).

### 2.2. mRNA Expression Profile of hiPSC-Derived Neurons

**Figure 2:**
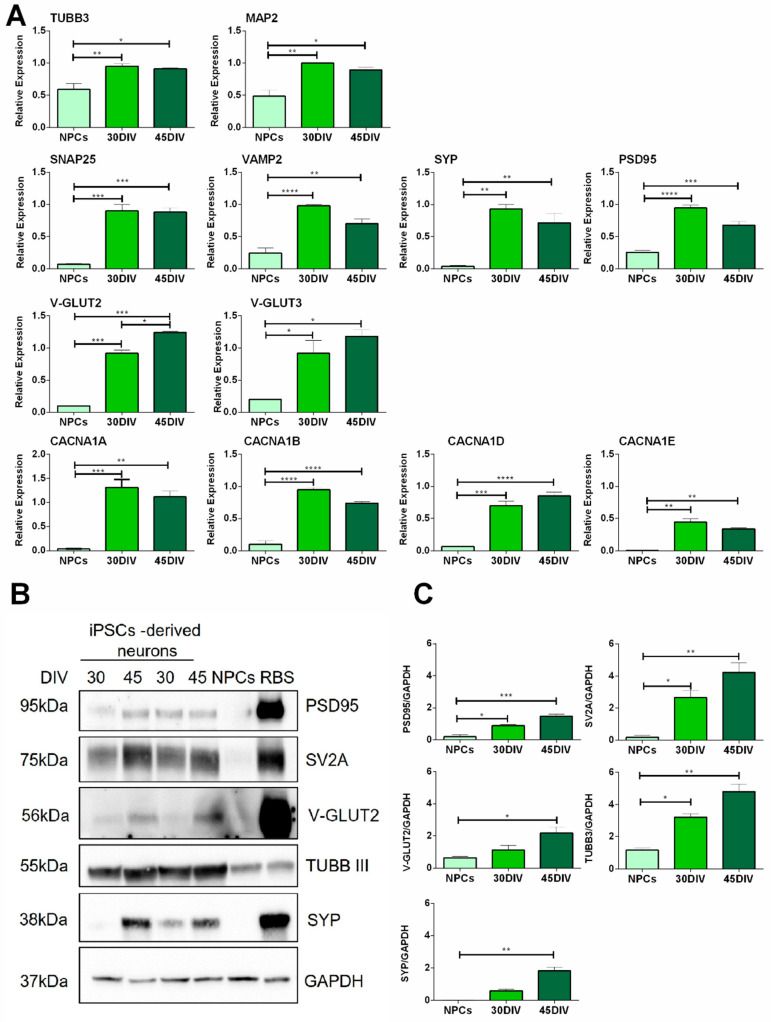
hiPSC-derived neurons express neuronal markers. (**A**) Gene expression profiling of hiPSC-derived neurons by qRT-PCR showing upregulation of neuronal markers in neurons at mature stages (30–45 days of differentiation in vitro (DIV)). Neuronal markers: Tubulin Beta 3 Class III (Tubb3), Microtubule-associated protein 2 (Map2); synaptic markers: Synaptosome Associated Protein 25 (Snap25) (pre-synaptic), Vesicle Associated Membrane Protein 2 (Vamp2) (pre-synaptic), Synaptophysin (Syp) (pre-synaptic), Discs Large MAGUK Scaffold Protein 4 (Postsynaptic density protein 95) (Psd95) (post-synaptic); Vesicular Glutamate Transporters type: V-Glut2, V-Glut3; Calcium Voltage-Gated Channel Subunits Alpha1A,B,D,E: Cacna1a, Cacna1b, Cacna1d, Cacna1e. Bar graphs show the mean ± SEM of relative expression; at least *n* = 3 replicate for each group. * *p* < 0.05, ** *p* < 0.01, *** *p* < 0.001, **** *p* < 0.0001, by one way ANOVA, Bonferroni’s multiple comparisons tests. NPCs were used as negative control. (**B**) Representative images of western blot scans showing total PSD95, SV2A, V-GLUT2, TUBB3, SYP, and Glyceraldehyde-3 phosphate dehydrogenase (GAPDH) protein levels from hiPSC-derived neurons (30–45 DIV), NPCs and rat brain synaptosomes (RBS). (**C**) Western blot quantification of PSD95, SV2A, V-GLUT2, TUBB3, and SYP. Values were normalized on GAPDH expression. Bar graphs show the mean ± SEM of relative expression; at least *n* = 3 replicate for each group. * *p* < 0.05, ** *p* < 0.01, *** *p* < 0.001, by one way ANOVA, Bonferroni’s multiple comparisons tests. NPCs were used as a negative control.

We compared the expression profile of specific neuronal markers in NPCs and hiPSC-derived neurons at 30 and 45 DIV by RT-qPCR (Figure 2). We first tested neuronal markers Tubulin Beta 3 Class III (Tubb3) and Map2 and found significantly increased expression in hiPSC-derived neurons (Figure 2). Next, we evaluated the expression of specific pre- and post-synaptic markers, underlying the development of synaptic activity. Synaptosome Associated Protein 25 (Snap25), Vesicle Associated Membrane Protein 2 (Vamp2), Synaptophysin (Syp), and Discs Large MAGUK Scaffold Protein 4 (Postsynaptic density protein 95) (Psd95) were all upregulated in the hiPSC-derived neurons at 30 and 45 DIV compared to NPCs (Figure 2). Moreover, we showed upregulation of the vesicular glutamate transporters, V-Glut2 and V-Glut3, indicating the prominent excitatory phenotypes of the differentiated cells (Figure 2). As critical regulators of calcium influx involved in the release of neurotransmitters, we assessed the expression profile of the main voltage-dependent calcium channels alpha subunits, such as Alpha1A (Cacna1a), Alpha1B (Cacna1b), Alpha1D (Cacna1d), Alpha1E (Cacna1e) and found a significant upregulation in hiPSC-derived neurons compared to NPCs.

The expressions of Psd95, Synaptic vesicle glycoprotein 2A (Sv2a), V-Glut2, Tubb3, and Syp, were evaluated also at the protein level, as illustrated in the Figure 2B,C. NPCs and rat brain synaptosomes were used as a negative and a positive control, respectively.

### 2.3. Morphological Analysis of hiPSC-DerivedNeurons

**Figure 3:**
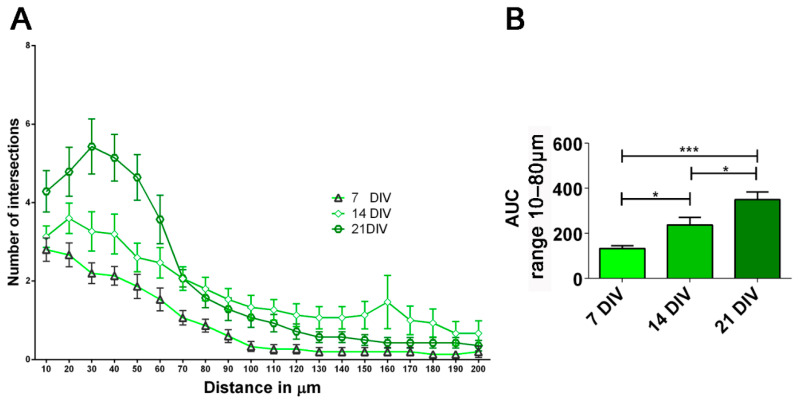
Morphological analysis on hiPSC-derived neurons. (**A**) Sholl analysis showing an increase in branching at the analyzed time points: 7, 14, 21 DIV. (**B**) Area under the curve (AUC) from 10 µm to 80 µm graph showing significant increment of dendritic outgrowth at 7, 14, 21 DIV. Histogram values were means ± SEM. * *p* < 0.05, *** *p* < 0.001 by one way ANOVA, Bonferroni’s multiple comparisons tests.

To explore in detail the differentiation process, we evaluated neuronal morphology by analyzing the complexity of the dendritic arborization at different time points [39] (7, 14, and 21 DIV). As shown in Figure 3, the number of dendritic branches analyzed by Sholl Analysis grow from 7 to 14 and 21 DIV, indicating a progressive increase of the dendritic complexity during the developmental stages. The total neurite length also showed a tendency to increase between the time points but did not reach the statistical significance (data not shown). The complexity of the dendritic tree gradually decreased as the distance from soma increased, consistent with the growth of the axon process [40,41].

### 2.4. Analysis of NMDA, mAChRs and 5HT Receptors Expression in hiPSC-DerivedNeurons

**Figure 4:**
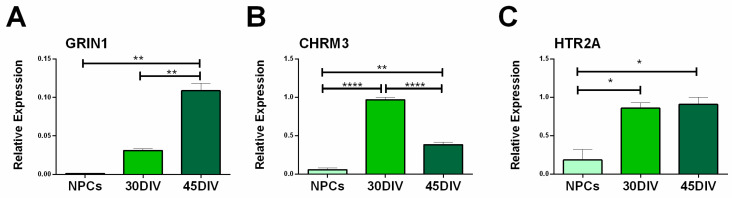
Expression of N-methyl-D-aspartate (NMDA) receptor, Muscarinic acetylcholine receptor (mAChR) and 5-Hydroxytryptamine receptor (5HTR) in NPC and hiPSC-derived neurons. Bar graphs showing RT-qPCR results for Glutamate Ionotropic Receptor NMDA Type Subunit 1 (Grin1; (**A**)), Cholinergic Receptor Muscarinic 3 (Chrm3; (**B**)), and 5-Hydroxytryptamine Receptor 2A (Htr2a; (**C**)) at 30 and 45 DIV. All receptors showed upregulation in hiPSC-derived neurons compared to NPCs. Histogram bars represent the mean values ± SEM of relative expression, at least *n* = 3 replicated for each group. * *p* < 0.05, ** *p* < 0.01, **** *p* < 0.0001, by one way ANOVA, Bonferroni’s multiple comparisons.

We further investigated the maturity state of hiPSC-derived neurons by studying the expression of N-methyl-D-aspartate receptor (NMDA), Muscarinic acetylcholine receptors (mAChRs), and the receptor for serotonin, 5-Hydroxytryptamine Receptor 2A (Htr2a) at 30 and 45 DIV by RT-qPCR. We focused on Glutamate Ionotropic Receptor NMDA Type Subunit 1 (GRIN1), a critical subunit of NMDA receptors involved in the plasticity of synapses and in the activation of calcium-dependent signaling pathways [42,43,44,45]. We found that hiPSC-derived neurons, and not NPCs, showed significant Grin1 mRNA expression, peaking at 45 DIV (Figure 4A). Among mAChRs, we evaluated the expression of Cholinergic Receptor Muscarinic 3, Chrm3, and found a significant upregulation in hiPSC-derived neurons at both the time points, with respect to NPCs (Figure 4B). The expression of Chrm3 declines with the progression of the differentiation. hiPSC-derived neurons showed also higher expression levels of the serotonin receptor Htr2a at 30 and 45 DIV compared with the NPCs (Figure 4C).

### 2.5. Immunofluorescence Evaluation of the Synaptic Complex in hiPSC-DerivedNeurons

**Figure 5:**
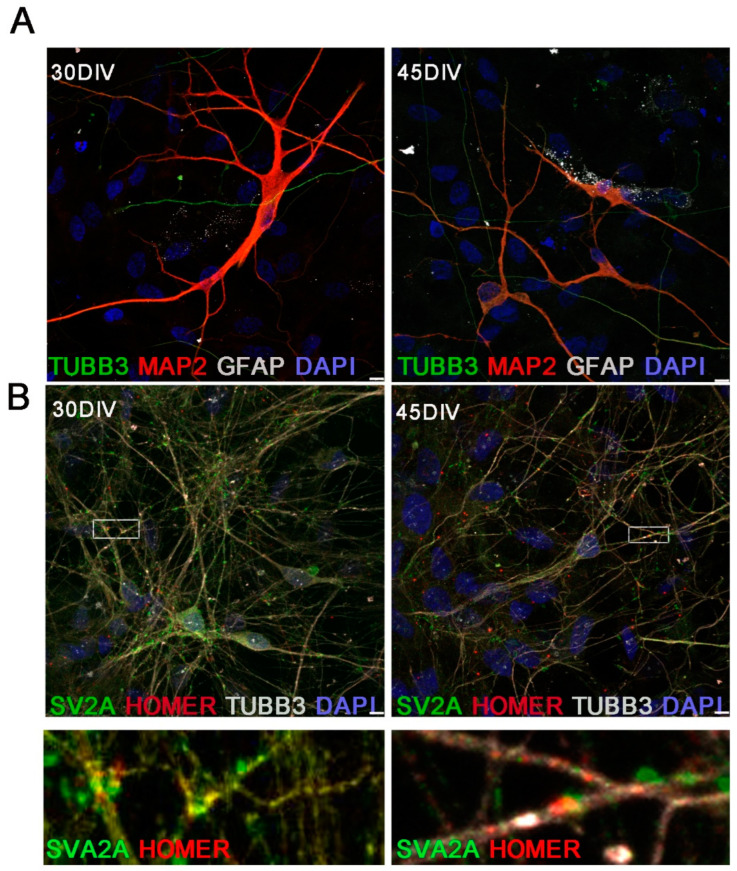
Expression of neuronal proteins in hiPSC-derived neurons. (**A**) Representative confocal images of hiPSC-derived neurons at 30–45 DIV showing expression of the neuronal markers TUBB3 and MAP2 and the astrocyte marker Glial Fibrillary Acidic Protein (GFAP). Scale bar 7.5 µm. (**B**) Synaptic proteins SV2A (green) and HOMER (red) co-localized at 30–45 DIV. Scale bar 7.5 µm. The rectangle frames are the regions of interest shown below at higher magnification.

To assess the efficiency and homogeneity of the neuronal differentiation, we performed immunofluorescence analysis by confocal microscopy (Figure 5A). Immunolabelling of hiPSC-derived neuronal cultures at 45 DIV showed positivity for astrocyte marker Glial Fibrillary Acidic Protein (GFAP) in 3% of DAPI-positive cells. Neuronal markers TUBB3 and MAP2 were detected in 58% (Figure 5A) of the total cells. In addition, detection of the pre-synaptic protein SV2A and the post-synaptic protein HOMER confirmed the presence of synaptic boutons at 30 and 45 DIV (Figure 5B and Figure S2).

### 2.6. hiPSC-DerivedNeurons Release Glutamate in Response to Depolarization

**Figure 6:**
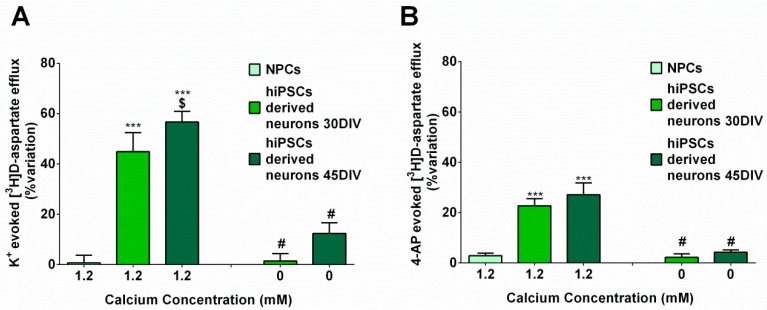
Exocytotic (Ca^2+^-dependent) release of glutamate from hiPSC-derived neurons at 30 and 45 DIV. (**A**) K^+^-evoked glutamate release from hiPSC-derived neurons in superfusion at 30 and 45 DIV. Data are expressed as mean ± SEM from three to seven different experiments. (**B**) 4AP-evoked glutamate release from hiPSC-derived neurons in superfusion at 30 and 45 DIV. Data are expressed as mean ± SEM from three to seven different experiments. (**A**) *** *p* < 0.001 vs. effect in NPCs, # *p* < 0.001 vs. K^+^ in 1.2 mM Ca^2+^ at the same day of differentiation and $ *p* < 0.05 vs. K^+^ in 1.2 mM Ca^2+^ at 30 DIV; (**B**) *** *p* < 0.001 vs. 4-AP in NPCs, # *p* < 0.001 vs. 4-AP in 1.2 mM Ca^2+^ at the same day of differentiation (one way ANOVA followed by Bonferroni’s post hoc test). For other experimental details see Materials and Methods.

**Figure 7:**
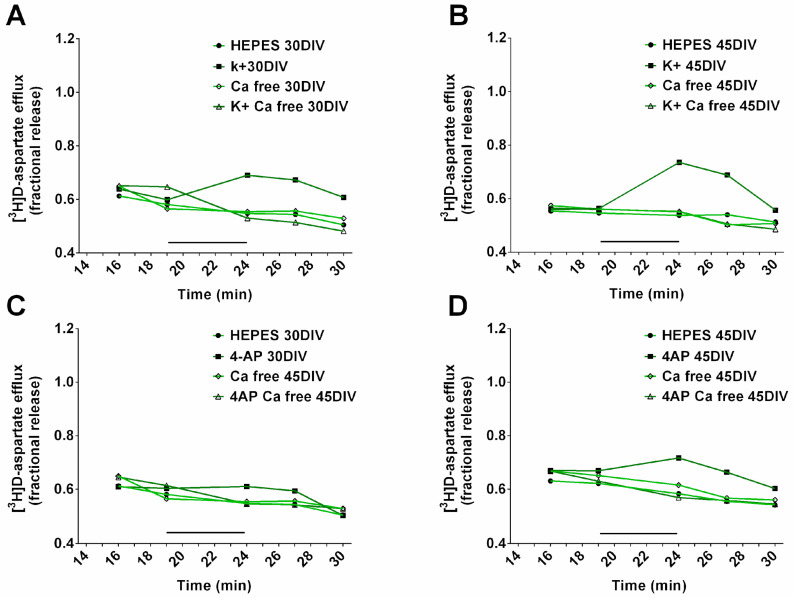
Time courses for the evoked glutamate release. (**A**,**B**). K^+^-evoked glutamate release from hiPSC-derived neurons in superfusion. Representative time-courses for the release of tritium from hiPSC derived neurons at 30 (**A**) and 45 (**B**) day of differentiation are shown. K^+^ was added (3 min; black bar) during superfusion. (**C**,**D**). 4-AP-evoked glutamate release from hiPSC-derived neurons in superfusion. The representative time courses for the release of tritium from hiPSC-derived neurons at 30 (**C**) and 45 (**D**) days of differentiation are shown. 4-AP was added (3 min; black bar) during superfusion. For other experimental details see Materials and Methods.

We next assessed the glutamate-releasing properties of the neuronal cultures by observing biochemical assays in different conditions. The mean [^3^H]D-aspartate release from NPCs amounted to 4.45 +/− 0.805 (*n* = 5), and the release from hiPSC-derived neurons to 0.68 +/− 0.0253 (*n* = 4) and to 0.73 +/− 0.0330 (*n* = 7) after 30 or 45 DIV, respectively. The mean [^3^H]D-aspartate release from hiPSC-derived neurons in the absence of extracellular Ca^2+^ amounted to 0.59 +/− 0.1440 (*n* = 3) and to 0.65 +/− 0.007 (*n* = 4) after 30 or 45 DIV, respectively, which did not differ from the corresponding values in the presence of extracellular Ca^2+^. Depolarization with high KCl or with 4-Aminopyridine (4-AP) evoked [^3^H]D-aspartate release from hiPSC-derived neurons at both 30 or 45 DIV, but was completely ineffective on NPCs (Figure 6A,B). Notably, in the absence of extracellular calcium, the depolarization-evoked release from hiPSC-derived neurons was abolished. Representative time-course for [^3^H]D-aspartate release from hiPSC-derived neurons in response to K^+^ or 4-AP depolarization, in the presence or in the absence of extracellular Ca^2+^ are shown in Figure 7A and Figure 7B, respectively.

## 3. Discussion

The generation of functional human neurons through iPSCs represented a breakthrough for the study of neurological disorders, neurodevelopmental disorders, and neuropsychiatric disorders, allowing for the study of otherwise inaccessible cells [46]. In recent years, multiple protocols have been described to achieve neuronal differentiation of mixed or specific neuronal subtypes [47,48,49,50,51]. To explore glutamate release properties of hiPSC-derived neurons, our study focused on excitatory neurons. In physiological conditions, these neurons differentiate from a population of neuroprecursors located in the cortical ventricular zone that migrate in the upper layers of the cortex during the development stages. In vitro, the differentiation process is largely recapitulated by iPSCs maturating into neuronal cells, using specific protocols. The most common protocol of differentiation includes the generation of neuroprecursor cells (NPCs) as intermediated cellular populations, through the administration in the culture media of dual inhibition of SMAD signaling factors [36,38,52]. In this study, we used the inhibition of SMAD pathways to obtain neuronal differentiation and neuronal rosettes formation, as a renewal reservoir of the NPCs [53,54,55]. In the present work, we successfully established a novel culture system to obtain hiPSC-derived neurons. In standard approaches, differentiating neurons are co-cultured with rat or mouse astrocytes to achieve full maturation. In our protocol, NPCs were plated in a feeder-free state and small molecules and BDNF, GDNF, and RA were added into the medium to generate a population of cortical excitatory neurons in 30–45 DIV. Therefore, the protocol emerges as a powerful tool to assess the specific contribution of neuronal population in physiological and pathological processes, such as those involving secretory and intercellular signaling in the absence of astrocyte confounding background. In addition, our method may find future clinical applications in regenerative medicine by abolishing animal-derived products.

### 3.1. Feeder-Free hiPSC-DerivedNeurons Express Neuronal Markers and Morphology

The hiPSC-derived neurons at mature stages displayed neuronal transcriptional networks. First, we observed the onset of neural differentiation by the expression of neuronal cell markers, the neuronal microtubule marker Tubb3 and the dendrite marker Map2 in the NPCs population. The increase of the expression of Tubb3 and Map2 in hiPSC-derived neurons at 30–45 DIV indicated the efficient conversion of NPCs to neuronal populations [56,57]. We confirmed the maturation of hiPSC-derived neurons from NPCs by the expression of synaptic genes Syp and Snap25, encoding key components of machinery for exocytotic release of neurotransmitters at the presynaptic active zones, of the vesicle-associated protein Vamp2, as well as of postsynaptic protein Psd95, indicating the ability of hiPSC-derived neurons to develop neurotransmitter-releasing nerve terminals. The presence of VGLUTs defines subsets of excitatory glutamatergic neurons [58]. NPCs neuronal differentiation was described to lead to the generation of hiPSC-derived neurons predominantly expressing V-Glut1 [59], while in this work we demonstrated the expression of V-glut2 in our iPSC-derived neurons. This finding could suggest high levels of ventral hindbrain markers in our NPCs populations [60]. In any case, expression of genes for glutamate vesicular transporters VGLUTs suggested maturation of glutamate-releasing exocytotic vesicles.

Additionally, morphological analysis of hiPSC-derived neurons on Geltrex coating confirmed the physiological neuronal development process, displaying an increase in neurite crossings at the developmental stages 7, 14, 21 DIV. [61]. In fact, the reduction of number of dendrites far from the soma is indicative for the growth of the axon process [40,41].

Finally, we showed significant expression of genes encoding for diverse voltage-gated Ca^2+^ channel subunits, required to activate Ca^2+^-dependent exocytotic release of the neurotransmitter in proximity of the active zone ([62] and references therein), consistent with maturation of the machinery for exocytotic neurotransmitter release (for discussion, see below the Section 3.2). The hiPSC-derived neurons at mature stages also expressed receptors for neurotransmitters such as NMDA receptors for glutamate, muscarinic receptors for acetylcholine, and 5-HT2 receptors for serotonin. Therefore, they appeared equipped with receptors potentially allowing them to sense the extracellular signals. This appears of critical importance, as acquiring the characteristics of a mature neuron specialized for intercellular communication requires the ability both to sense extracellular signals through the expression of receptors for neurotransmitters, and to send signals (i.e., to release neurotransmitters). In particular, the neurotransmitters glutamate, acetylcholine, and serotonin play relevant roles in neuronal development. The NMDA receptors for glutamate are well known to be involved in neuron development [45]; notably, we report that the NMDA subunit 1, involved in calcium-dependent signaling and synapse plasticity [42,43,44,45], was expressed in hiPSC-derived neurons. Muscarinic acetylcholine receptors are involved in cell proliferation and neuronal differentiation also occurring early prior of the onset of neurogenesis ([63] and references therein); interestingly, and consistent with such early roles for cholinergic signaling through muscarinic receptors, we observed an early expression of these receptors confirmed their prominent role in the early stage of neurogenesis [64]. On the other hand, serotonin is a well-known signal implicated in cell proliferation, synaptogenesis and apoptosis, and cortical development [65]. Recent evidence indicates that serotonin promotes basal progenitor cell proliferation in an evolutionary relevant manner through 5-HT2A receptors, suggesting that this mechanism may have contributed to neocortex expansion in humans [66]. Higher expression levels of the 5-HT2A receptor at 30 and 45 DIV confirm the cortical fate of differentiated neurons [67]. Immunofluorescence analysis of synaptic boutons at mature stages showed co-localization between SV2A, a transmembrane protein in secretory vesicles critical for Ca^2+^-dependent exocytosis in central neurons, and postsynaptic excitatory marker HOMER1 [68]. The progressive neuronal maturation is also confirmed by the analysis of the neurite length, branching, and complexity of the dendritic arborization.

### 3.2. hiPSC-DerivedNeurons Release Glutamate in Response to Depolarization

The ability of hiPSC-derived neurons to send signals was studied by measuring the release of [^3^H]D-aspartate, the non-metabolizable analogue of glutamate. By taking advantage of a set-up specifically designed to study the release of neurotransmitters in superfusion from nerve terminals, cells, or slices [69] we could directly measure the neurotransmitter release and appreciate the ability of cell depolarization to evoke it as well as its dependency on the extracellular Ca^2+^ availability, a prerequisite for vesicular exocytotic release. The finding that hiPSC-derived neurons obtained from fibroblasts under feeder-free conditions were able to release glutamate in a Ca^2+^-dependent way in response to depolarization appears relevant, as it would contribute to bridging the gap between the demonstration of the expression of neuronal transcriptional networks and direct demonstration of specific neuron functioning. According to our knowledge, Ca^2+^-dependent release of a putative neurotransmitter was never shown to be evoked by the depolarization of human-induced neuron-like cells. Neurons differentiated from hiPSCs from fibroblasts were able to release the neurotransmitters dopamine and catecholamines upon K^+^ depolarization [70,71,72,73]; differentiation from Parkinson’s disease patients was also successful [70,72]. However, Ca^2+^-dependency and modes for release were however not further investigated. Notably, it was recently reported that human fibroblasts could be efficiently and directly reprogrammed into glutamatergic neuron-like cells (by exposing cells to a combination of small molecules) [74]. These neuron-like cells exhibited mature firing patterns at electrophysiological analysis and appeared to connect in functional synapses through glutamatergic signaling [74]. Nevertheless, direct demonstration of the release of glutamate was not obtained. Notably, the ability of hiPSC-derived glutamatergic neurons (expressing VGLUTs) to connect in neuron networks was also reported by recording the collective behavior of the network on multielectrode assays devices [75]. In addition, hiPSC-derived neurons from schizophrenia patients showed synaptic release deficits when compared to neurons from healthy controls, as indicated by altered synaptic vesicle release upon depolarization [76]. The ability of human embryonic stem cells to differentiate into neurons releasing glutamate has been reported. In fact, cyclopamine treatment of human embryonic stem cells was found to increase the number of neurons expressing the VGLUT and the amount of glutamate released in the medium [77]. Furthermore, glutamate was suggested to be released upon light stimulation of Channelrhodopsin-2(R2)-expressing glutamatergic neurons derived from human embryonic stem cells; the stimulation was also found able to increase intracellular Ca^2+^ levels [78]. In these cases, however, no direct demonstration of glutamate release was reported. Here we report, for the first time to our knowledge, that human somatic cells can be induced towards a mature neuronal fate, exhibiting specific neuronal features and releasing [^3^H]D-aspartate, the non-metabolizable analogue of the neurotransmitter glutamate in a Ca^2+^-dependent manner upon depolarization. In fact, neuron-specific features include the ability to communicate through chemical signals, the neurotransmitters, by expressing receptors to receive neurotransmitter signals and release neurotransmitter signals in a Ca^2+^-dependent way. Among the neurotransmitters, glutamate is primarily involved in physiological signaling being involved in neuron differentiation during development and in neuron plasticity, learning, and memory in adult life [79,80]. On the other hand, dysregulated glutamate transmission is crucially involved in neuron damage in acute pathological conditions such as stroke or ischemia, as well as in chronic neuron damage in neurodegenerative diseases [81]; indeed, endpoints related to glutamatergic transmission are of primary relevance in the developmental neurotoxicity adverse outcome pathways [82]. Accordingly, a great deal of interest is focused on human-induced pluripotent stem cells towards glutamatergic neurons as a platform for mechanistic assessment of excitotoxicity or neurotoxicity, as seizure models, or in drug discovery [83,84,85]. It must be remembered that modes for neuronal communication through glutamatergic signaling are various, including exocytotic Ca^2+^-dependent vesicular glutamate release and Ca^2+^-independent modes, such as the function of transporters including the excitatory amino acid transporters [86,87] or the cystine-glutamate exchange transporter [88] and the efflux through receptor channels or accessory proteins [89,90]. Among these modes, the most specific neuronal mode for glutamate release is the exocytotic one, involving Ca^2+^-dependent activation of glutamate release from vesicles which were loaded with glutamate through specific VGLUTs [58]. The expression of VGLUTs appears therefore of primary importance in hiPSC-derived neurons and consistent with the appearance of vesicular exocytotic glutamate release. We are aware that VGLUTs cannot mediate the transport of [^3^H]D-aspartate into the synaptic vesicles [91,92]; the appearance of the VGLUTs is, nevertheless, indicative of the presence of a releasable L-glutamate pool. Moreover, aspartate has been shown to be accumulated in synaptic vesicles [93], presumably by an as-yet unidentified transporter [94,95] and the release of [^3^H]D-aspartate can, therefore, be used as a marker of neurotransmitter glutamate release. The upregulated expression of voltage-gated Ca^2+^ channel subunits, in particular of the subunit Alpha1A (encoded by *CACNA1A*), the specific pore-forming structure in P/Q-type Ca^2+^ channels and of Alpha1B (encoded by *CACNA1B*), the pore-forming subunit of an N-type Ca^2+^ channel which controls neurotransmitter release from neurons [62], also fits with vesicular Ca^2+^-dependent glutamate release in hiPSC-derived neurons. Notably, the voltage-dependent N-type and P/Q type Ca^2+^ channels are strategically located at the active zones in nerve terminals and their functioning is required for depolarization-evoked Ca^2+^ entry and vesicle fusion ([62] and references therein).

In summary, behind the displacement of neuronal transcriptional networks typical of glutamatergic neurons, including expression of pre- and post-synaptic glutamatergic markers such as VGLUTs and PSD95 the hiPSC-derived neurons behaved as glutamatergic neurons. Indeed, they were able to respond to high K^+^ or 4-AP depolarization by releasing glutamate. Expression of VGLUT was consistent with the glutamatergic nature of the neurons, and with the ability of depolarization to evoke release from the glutamatergic vesicles in a way dependent on the availability of extracellular Ca^2+^ and therefore on Ca^2+^ entry. Expression of the genes for subunits of voltage-dependent Ca^2+^ channels is consistent with the presence of the Ca^2+^ channels allowing depolarization-evoked Ca^2+^ entry and coupling to activation of vesicle fusion. Notably, the expression of V-Glut3 (present also in non-glutamatergic neurons; [96,97,98,99]) may indicate the ability of this protocol to differentiate hiPSCs into neurons co-releasing glutamate and other neurotransmitters such as acetylcholine, GABA, or serotonin; this may be subject to future research. The new methodological approach to achieve feeder-free neuronal differentiation from hiPSCs, avoiding inter-species contamination, may therefore represent a step-ahead not only in experimental neuroscience but also in neurotoxicology and neurodevelopmental toxicology as a platform for mechanistic assessment of excitotoxicity/neurotoxicity or for drug discovery [82,83,84,85]. In fact, our study is an attempt to develop a protocol allowing us to obtain a human neuron population suitable for in vitro modeling of brain disorders and new therapeutic approaches against the disorders.

Nevertheless, we have to bear in mind some limitations of the study. In particular, evidence for the ability to release a neurotransmitter in exocytotic vesicular mode in response to depolarization (the specialized mode for chemical signal release from neurons) must be considered only a starting point for assessing the cell ability to achieve a fully mature neuronal fate, paving the way to further investigations on the ability to integrate in networks, and to respond to network electrical stimulation. Another limitation of our study could be the lack of direct identification of morphologically developed synapses, i.e., by electron microscopy, and of synapse characterization, e.g., by mass synaptometry [100]. Our study might be enhanced by an in-depth analysis of the synaptic structures through electron microscopy and mass synaptometry, which will allow the assessment of maturation of hiPSC-derived neurons and synapses, and comparison of the synapse molecular signature in hiPSC-derived neuron with that of mature mammal neurons. Such a new technology has been applied to synaptosomal preparations [100,101] and might as well be applied to purified nerve terminals isolated from central nervous system (CNS) regions [69,90,102,103], or from hiPSC-derived neurons. Comparison of the molecular signature of nerve terminals from hiPSC-derived neurons and of the nerve terminals from rodents [69,90,102,103] or human [100,101] CNS regions might contribute to characterization and assessment of full maturation of the hiPSC-derived neurons. Indeed, the new technology for smaller size events has already opened a field for studying deep molecular signatures of neurodegenerative diseases such as Alzheimer’s or Parkinson’s disease [101,104]. Further investigation on in vitro feeder-free hiPSC-derived neurons from patients will provide further insight. In fact, our feeder-free methodology might be further developed and applied for modeling neurodegenerative disease in vitro with patients hiPSC-derived neurons [105], possibly allowing drug screening for personalized medicine.

## 4. Materials and Methods

### 4.1. Generation and Maintenance of Human Induced Pluripotent Stem Cells (hiPSCs)

The hiPSCs were generated from dermal fibroblasts. Skin biopsies were performed upon informed consent using the punch biopsy procedure and fibroblasts were cultured in RPMI (Gibco^TM^, Thermo Fisher Scientific, Monza, Italy) supplemented with 20% FBS, 2 mM LGlutamine, and 1% Penicillin/Streptomycin. Fibroblasts were obtained from the “Cell Line and DNA Biobank from Patients affected by Genetic Diseases” (IRCCS G. Gaslini, Genova, Italy), a member of the Telethon Network of Genetic Biobanks (project no. GTB12101). The study was supported by the local Ethical Committee of the G. Gaslini Institute and approved by the Ethic Committee of the Liguria Region: CE Regionale sez.1 (n°8/2015, 14 September 2015). Cells at low passages were reprogrammed by using the non-integrating Sendai virus CytoTune™-iPS 2.0 reprogramming Kit (Life Technologies, Monza, Italy) under feeder-free conditions according to the manufacturer’s instructions. Clones appeared after 25 days. At least 20 single colonies for each genotype were isolated by manual picking and maintained on recombinant human truncated Vitronectin (VTN-N)-coated plates in ESSENTIAL-8 culture medium (all from Life Technologies). Culture medium was refreshed every other day and cells were passaged using Versene solution every 3–5 days. Each clone was expanded separately as cell lines. At the 15th passage, clones were tested for the expression of pluripotency markers by qRT-PCR and IF. For in vitro differentiation into cells of all three germ layers, confluent undifferentiated hiPSCs were incubated in 1 mg/mLcollagenase IV (Life Technologies) for 20 min at 37 °C and transferred to 100 mm low attachment plates in EBs medium (DMEM/F12 supplemented with 20% knockout serum replacement, 1 mM glutamine, 1% non-essential amino acids, 1% penicillin/streptomycin, and 0.55 mM β-mercaptoethanol from Thermo Fisher Scientific). All hiPSC lines were mycoplasma-free, based on tests by EZ-PCR Mycoplasma Test Kit (BI Biological Industries, Resnova, Rome, Italy). At least two clones were raised for the following experiments.

### 4.2. Array-CGH Assay

Analysis of CNVs has been performed by Array-CGH using Human Genome CGH Microarray kit 8 × 60 K (AgilentTM Technologies, Milan, Italy) following to manufacturer’s protocol. Genomic DNA from parental fibroblasts and iPSCs clones were labeled with Cyanine-3 and Cyanine-5 and competitively hybridized on a CGH-array. Data were analyzed using Cytogenomics software (Genome coordinates are shown according to human genome build GRCh37/hg19) with the following analysis settings: aberration algorithm ADM-2; threshold 6.0; window size 0.2 Mb; filter 4 probes; and DLRS < 0.25.

### 4.3. Feeder-Free Differentiation of hiPSC Clones into Neurons

For neuronal differentiation, hiPSCs were detached from the culture plate and forced to aggregate into Embryoid Bodies-like structures, using the AggreWell™800 Plates (STEMCELLTechnologies Inc., Cologne, Germany). EBs were maintained in STEMdiff™ Neural Induction Medium + SMADi + 10 µM Y-27632 (STEMCELLTechnologies Inc.) for 4 days. At day 5, EBs were plated on Geltrex-coated plates in STEMdiff™ Neural Induction Medium + SMADi medium. After 6 days, EBs spread out and neural rosettes should be clearly visible. Neural rosettes were selected with STEMdiff™ Neural Rosette Selection Reagent and plated on Geltrex-coated plate. Selected rosettes attached, and NPC outgrowths formed a monolayer between the clusters. After a few days, neuroprecursors cells (NPCs) were ready for passage on Geltrex-coated plates and were maintained with STEMdiff™ Neural Progenitor Medium (STEM CELL Technologies Inc.). Finally, to differentiate NPCs into functional cortical neurons, human cells were grown in feeder-free conditions and plated on 18mm-round glass coverslips coated with Geltrex (Life Technologies). Cells were maintained in Neurobasal-A medium supplemented with L-glutamine (2 mM), N2 (1:100), B27 (1:50), BDNF (10 ng/mL) GDNF (10 ng/mL) (all from Life Technologies), and retinoic acid (1 μM; Sigma-AldrichS.r.l., Milan, Italy) to obtain a mixed network of excitatory and inhibitory neurons [49,106]. Half of the medium will be replaced every other day during continuous culturing. At 30–45 days of in vitro differentiation (DIV) iPSC-derived neurons will reach a state showing mature neuronal markers, with a mixed population of cortical inhibitory and excitatory neurons. Moreover, our cells will reach a mature expression of post and presynaptic markers such as SV2A, HOMER.

### 4.4. qRT-PCR

Total mRNA was extracted with the RNeasy mini kit (Qiagen, Milan, Italy) according to the manufacturer’s instructions. Quality and quantity of RNA were analyzed using a NanoDrop spectrophotometer (NanoDrop Technologes, Inc, Wilmington, DE, USA). The cDNA was synthesized from 250 ng of total RNA with the iScript cDNA Synthesis Kit (Bio-Rad Laboratories, Hercules, CA, USA). Each RNA sample was controlled for genomic DNA contamination without reverse transcriptase addition into a cDNA synthesis mixture. All primers are listed in Supplementary Table S1. qRT-PCR was performed in triplicate with the 2 Sso Fast EvaGreen Supermix in the CFX96 Real-Time PCR Detection System (Bio-Rad Laboratories). In brief, the 15 µL PCR mixture contained diluted 1:5 cDNA and 0.5 µmol of each primer. Relative expression was calculated based on the 2−ΔΔCt method [107] by normalizing data to the geometric mean of three housekeeping transcripts (Glyceraldehyde-3 phosphate dehydrogenase (Gapdh), Peptidylprolyl isomerase A (Ppia), Ribosomal Protein L13A (Rpl13a)) using the Bio-Rad CFX Maestro software (Bio-Rad Laboratories).

### 4.5. Western Blot

Cells were lysed in lysis buffer (NaCl 150 mM, EDTA 1 mM, TrisNaCl 50 mM pH 7.4, and 1% Triton X-100) supplemented with protease inhibitors cocktails (Sigma-Aldrich). Proteins were quantified by DC protein assay (Bio-Rad Laboratories). An amount of 40 µg of total protein was separated on 4–15% Mini-PROTEAN gel (Bio-Rad Laboratories). Gels were transferred on to a nitrocellulose membrane using Trans-Blot Turbo Transfer System and Trans-Blot Turbo Mini 0.2 µm Nitrocellulose Transfer Pack (Bio-Rad Laboratories). Membranes were blocked with 5% milk in Tris Buffered Saline 1X, 0.05% Tween 20, and after were incubated with the following primary antibodies: anti-PSD95 (SYSY 124 011, 1:1000; SYSY, Goettingen, Germany,), anti-SV2A (SYSY 119 011, 1:1000), anti-TUBB3 (Sigma-Aldrich T2200, 1:1000), anti- HOMER11 (SYSY 160 011, 1:1000), anti-SYNAPTOPHYSIN-1 (SYSY 101 101, 1:1000), anti- VGLUT2 (SYSY 135 421, 1:1000), and anti-GAPDH (sc:47724, 1:1000, Santa Cruz Biotechnology, Dallas, TX, USA). All antibodies are listed in Supplementary Materials Table S2. Horseradish peroxidase (HRP)-conjugated secondary antibodies used were from Merck-Millipore, Milan, Italy (Donkey anti-rabbit, AP182P) and Dako (polyclonal Goat anti-mouse immunoglobulins HRP, P0447). Clarity™ Western ECL Substrate (Bio-Rad Laboratories) was used for the detection. Images were acquired by Uvitec Mini HD9 (Uvitec Cambridge, Cambridge, UK).

### 4.6. Immunocytochemistry

For hiPSCs, three germ layer cells and neuronal precursor cells staining was performed with PSC 4-MARKER ICC KIT, 3-GERM LAYER ICC KIT and NSC ICC KIT, respectively, (Thermo Fisher Scientific) according to the manufacturer’s instructions. For the characterization of hiPSC-derived neurons, cells were fixed with 4% PFA for 15min at room temperature, permeabilized with 0.1% Triton X-100 (Sigma-Aldrich) and blocked with 0.1% Triton X-100 with 5% FBS in PBS for 20 min. Samples were then incubated with primary antibodies at 4 °C o/n, followed by incubation with secondary antibodies for 1 h at room temperature. Antibodies were prepared in PBS containing 0.1% Triton X-100 and 5% FBS serum. Primary antibodies used for neuronal characterization: anti- HOMER 1 (SYSY 160 011, 1:100), anti-SV2A (SYSY 119 011, 1:100), anti-MAP2 (SYSY 188003, 1.200), anti-GFAP (Merck-Millipore, MAB360, 1:100), and anti-GLUT2 (SYSY 135 421, 1:1000). All antibodies are listed in Supplementary Materials Table S2. Fluorescent secondary antibodies (Alexa Fluor) were from Thermo Fisher Scientific. Coverslips were mounted by using Fluoromount-G (SouthernBiotech, Birmingham, AL, USA) containingDAPI to visualize nuclei. Images in epifluorescence were collected with an Axio Imager 2 Zeiss, ×40, ×60 objectives (Carl Zeiss S.p.A., Milan, Italy). Confocal images were acquired by using a 40× objective in a Leica TCS SP8 Confocal Laser Scanning Microscope (Leica Microsystems, Buccinasco, MI, Italy). For morphological analysis, hiPSC-derived neurons were labeled with a live dye NeuroFluor NeuO (STEM CELL Technologies Inc.) to follow neuronal process in three different time points: 7, 14, and 21 days of in vitro differentiation (DIV). Images were processed and analyzed using the free software ImageJ (https://imagej.nih.gov/ij/, accessed on 18 July 2022). Neurite length and Sholl analyses were performed using the ImageJ plugins NeuronJ and Sholl Analysis, respectively [108].

### 4.7. Assessment of Glutamate Release

The study of the release of glutamate from cells was performed by applying the method for measuring the release of neurotransmitters or gliotransmitters in superfusion. The up–down superfusion was applied to isolated nerve terminals or astrocyte processes [102] and with few modifications to astrocytes [109] and cells [89]. The method has been effectively used to study modes for the release of the neurotransmitter/gliotransmitter glutamate, allowing us to assess the vesicular mode [102], the reversal of the transmitter transporters [110], or the release through receptors, such as the purinergic P2X7 receptor for ATP [89] or through the recruitment of accessory molecules/channels [90]. We previously observed no difference in the behavior of endogenous glutamate or [^3^H]D-aspartate release from either cells [109], slices [111], or from nerve terminals or astrocyte processes prepared from rodent CNS regions [103], supporting the use of [^3^H]D-aspartate. Briefly, after incubation with non-metabolizable glutamate analog [^3^H]D-aspartate (0.25 μM, 30 min, 37 °C), cells were transferred to parallel superfusion chambers and then superfused with standard medium (0.5 mL/min; 37 °C) [102]. Starting at t = 33 min, we collected 3-min superfusate samples (fraction from B1 to B5); at t = 38 min, the depolarizing stimulus (1000 µM 4-AP or 50 mM K^+^; 6 min) was applied. To study the calcium dependency of [^3^H]D-aspartate release, the standard medium was changed to a Ca^2+^ free medium added with EDTA 0.5mM. In each experiment, at least one chamber was superfused with standard medium or with Ca^2+^ free medium and was used as a control chamber. The radioactivity in all the superfusate samples collected, and the radioactivity remaining in the cells at the end of superfusion was measured by liquid scintillation counting. The tritium fractional release in each collected sample (percentage of the total radioactivity at the onset of the sample collection) was used to evaluate the efflux of radioactivity and calculated according to the formula:

$$F_{x}=\frac{T_{x}}{T_{cell}+\sum_{k=x}^{n}T_{k}}\;\cdot 100$$

where:

*F_x_* = fractional release in the fraction *x*
*T_x_* = tritium content in the fraction *x*
*T_cell_* = tritium content in the cells at the end of perfusion
*n* = number of the fractions collected during perfusion
*1* ≤ *x* ≤ *n*

For each chamber the mean fractional tritium release in B1 and B2 fractions was taken as the 100% control value; tritium efflux in Bn fractions was measured as percent variation with respect to the control value. The 4-AP or K^+^-evoked tritium efflux was measured by subtracting the area under the curve of percent variations of the tritium fractional release in control chambers from the area under the curve of the percent variations in drug-treated chambers. In Figure 7, representative time courses of [^3^H]D-aspartatereleased in the collected samples in single experiments are shown.

### 4.8. Statistical Analysis

The normal distribution of experimental data was assessed using the D’Agostino-Pearson normality test. Data with normal distribution were analyzed by the unpaired Student’s *t*-test or one-way analysis of variance (ANOVA), followed by the Bonferroni’s multiple comparison tests. Non-normally distributed data were analyzed by the Kruskal–Wallis one-way analysis of variance on Ranks test followed by the Dunn’s multiple comparison tests. For release of [^3^H]D-aspartate statistical analysis was performed by commercial software using ordinary one-way ANOVA followed by Bonferroni post hoc test. Data are expressed as means ± standard error (SEM) of the number of experiments (*n*). A *p*-value < 0.05 was considered statistically significant.

## 5. Conclusions

In conclusion, our evidence indicates that the hiPSC-derived neurons obtained with a novel protocol displaced neuronal transcriptional networks typical of glutamatergic neurons and behaved as glutamatergic neurons. It is of note that they seem to acquire specific neuronal features, i.e., the ability to communicate through chemical signals, by expressing receptors to receive neurotransmitter signals, and by releasing neurotransmitter in a Ca^2+^-dependent way, consistent with maturation of the exocytotic mode of release in neurons. It is also of note that hiPSC-derived neurons are increasingly exploited to better understand defects in neurotransmission in central nervous system diseases such as the Alzheimer disease, Parkinson’s disease, Huntington’s disease, and familial schizophrenia to pave the way to true personalized treatments. In these patient-derived neurons, some evidence for dysfunction at synapse level or at the vesicle cycle for exocytotic release have been reported. Surprisingly, while data have been reported suggesting neurotransmission dysfunctions in hiPSC-derived neurons from patients, the neurotransmission was rarely approached by direct measurement of the release of the neurotransmitters. Direct measurement of the release of the transmitters, and investigation of the modes of release, would not only allow a step forward in the assessment of acquirement of mature neuron features but also help to a better understanding of the dysfunction at neurochemical and synaptic level in feeder-free hiPSC-derived neurons, also in disease models.

## Data Availability

Data are available upon request to the corresponding authors.

## Supplementary Materials

The supporting information can be downloaded at: https://www.mdpi.com/article/10.3390/ijms231810545/s1.

## Abbreviations

4-AP	4-Aminopyridine
AUC	Area under the curve
BDNF	Brain-Derived Neurotrophic Factor
bFGF	basic fibroblast growth factor
CACNA1	Voltage-dependent calcium channels Alpha1 subunit
ChR2	Channelrhodopsin-2
CHRM3	Cholinergic Receptor Muscarinic 3
CNS	Central Nervous System
DAPI	4′:6-diamidino-2-phenylindole
DIV	Days of differentiation in vitro
EBs	Embryoid bodies
FBS	Foetal bovine serum
GAPDH	Glyceraldehyde-3 phosphate dehydrogenase
GDNF	Glial cell-derived neurotrophic factor
GRIN1	NMDA Type Subunit 1
hiPSCs	Human induced pluripotent stem cells
HTR2A	5-Hydroxytryptamine Receptor 2A
IF	Immunofluorescence
KLF4	Kruppel Like Factor 4
mAChR	muscarinic acetylcholine receptors
MAP2	Microtubule-associated protein 2
NeuN	Neuronal nuclear protein
NMDA	N-methyl-D-aspartate receptor
NPCs	Neuroprecursors cells
OCT4	Octamer-binding transcription factor 4
PAX6	Paired box 6
PBS	Phosphate Buffered Saline
PCR	Polymerase Chain Reaction
PPIA	Peptidylprolyl isomerase A
PSD95	Discs Large MAGUK Scaffold Protein 4
RA	Retinoic acid
RBS	Rat Brain Synaptosome
RPL13A	Ribosomal Protein L13A
SMAD	Small mother against decapentaplegic
SNAP25	Synaptosome Associated Protein 25
SOX1 and 2	SRY-Box Transcription Factor 1 and 2
SSEA1	Stage specific embryonic antigen-1
SV2A	Synaptic vesicle glycoprotein 2A
SYP	Synaptophysin
TGF-b	Transforming Growth Factor-β
Tra1-60	T cell receptor alpha locus1-60
TUBB3	Tubulin Beta 3 Class III
VAMP2	Vesicle Associated Membrane Protein 2
VGLUT (2 and 3)	Vesicular glutamate transporters (type 2 and 3)
