# Supplementary material for: Vesicular Glutamate Release from Feeder-FreehiPSC-Derived Neurons
Source: Int J Mol Sci. 2022 Sep 11;23(18):10545. doi: 10.3390/ijms231810545 (PMC9501332; doi:10.3390/ijms231810545)
Supplement: Supplementary file 1 [file ijms-23-10545-s001.zip › ijms-1848724-supplementary.pdf]

Supplementary Materials Baldassari et al.

Title: Vesicular glutamate release from feeder-free hiPSC-derived neurons

Figure S1. Array CGH analysis

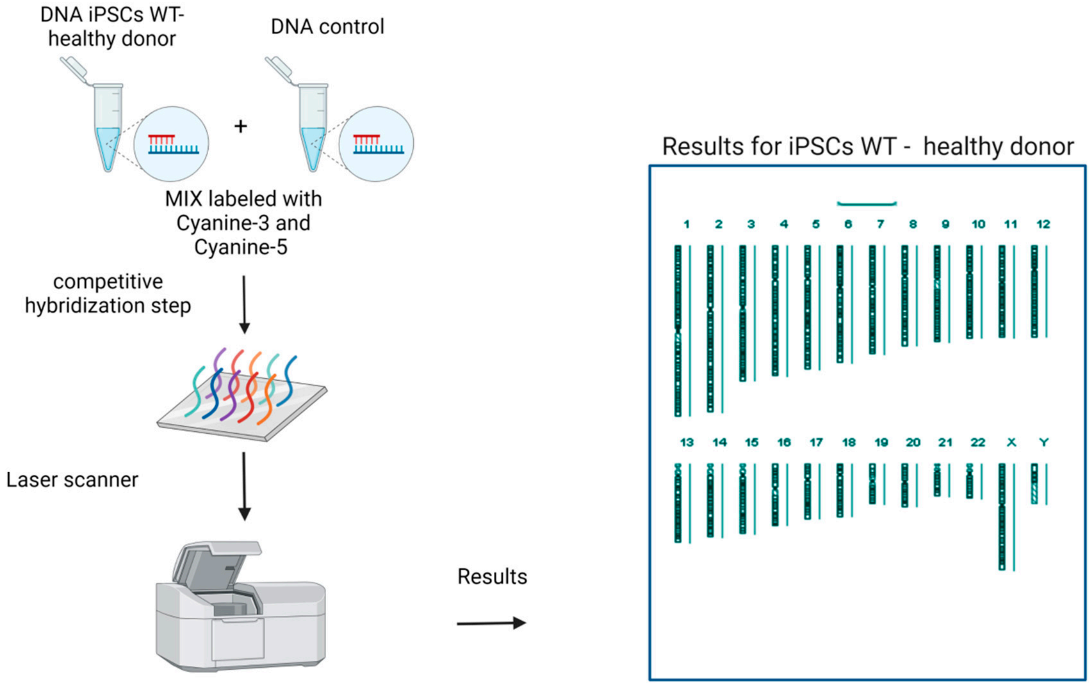

On the left: Schematic representation of array-CGH analysis DNAfrom iPSCs WT clone versus DNA control.

On the right: Array-CGH showed the complete absence of chromosomal rearrangements on iPSCs WT clone used.

Figure S2. **Immunofluorescence for synaptic proteins from different experimental group**

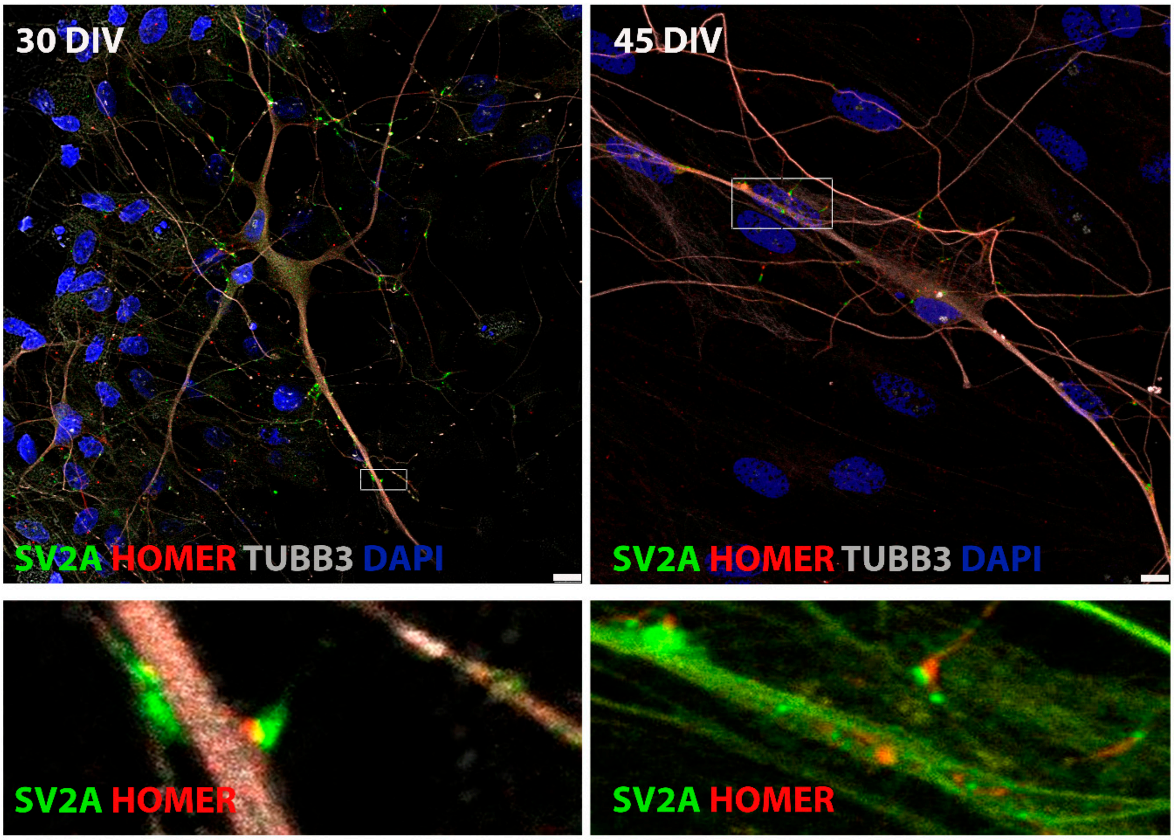

Figure S2. Representative images of expression of neuronal proteins in hiPSC-derived neurons, obtained from different experimental group. Synaptic proteins SV2A (green), HOMER (red) co-localized at synaptic sites at 30-45 DIV. Neuronal marker for microtubule element TUBB3 (gray). Total cells were stained with nuclear marker DAPI (blu). Scale bar 10 µm.

Table S1: Primers sequences

Gene Name	Forward	Reverse	Accession number
human NANOG	TGTCTTCTGCTGAGATGCCT	AATAAGCAGATCCATGGAGGA	NM_024865.3
human NESTIN	CAGAGGTGGGAAGATACGGT	AGCTCTGCCTCATCCTCATT	NM_006617
human SOX2	ACCAGCTCGCAGACCTACAT	CCTGCTGCGAGTAGGACAT	NM_003106
human SOX1	TCCTGGAGTATGGACTGTCCG	GAATGCAGGCTGAATTCGG	NM_005986
human PAX6	GATAACATACCAAGCGTGTCATCAATA	TGCGCCCATCTGTTGCT	NM_000280
human CACNA1A	AAACCCACCATCACCACCGA	GTCCCAAGCCCACGGTTTTC	NM_001127222
human CACNA1B	TGGAACTGACTTCGACCTGC	CTTGAGCACCACCTGCAAAC	NM_000718
human CACNA1D	TGAACATGGTCTTCACCGGG	TGCTGCCGATTACGATGAGG	NM_000720
human CACNA1E	CTGGCGAACGCTTTGGGA	TCACTACGCAGTCGAAGACG	NM_001205293
human MAP2	TGAACAAGAGAAGGAAGCCCAA	GAAGGTGGCAGATTAGCTGTTT	NM_002374
human TUBB3	AGCGTCTACTACAACGAGGC	GATGTCCAAAGGCCCCTGAG	NM_006086
humand PSD95	CATCCCTCCCTTTTCCCCAA	CTGCCTTCCACATGCCCC	NM_001369566
human SYP	AGTGTTCGCTTTCATGTGGC	GGCCATCTTCACATCGGACA	NM_003179
human VAMP2	CTGCACCTCCTCCAAACCTT	CAGCTCCGACAACTTCTGGT	NM_014232
human VGAT	CAGCTGCGAGGGTCATGAG	GAACGCGATGCAAGAAGGC	NM_080552
human VGLUT2	CCAGGAAAAGAGGGGCTAAA	CGGTGTCTTGCTTCTTCTCC	NM_020346
human VGLUT3	CTCCCCAAGCGTTACATCAT	ATTTCCACAATGGCAACTCC	NM_139319
human CHRM3	ATCGGCAACATCCTGGTAAT	GTCACAGGCCAAGTTCCCTA	NM_001375978
human GRIN1	GAAGCACGAGCAGATGTTCC	GTTGGGCTTGTGCGTGAC	NM_000832
human HTR2a	GGATTTACCTGGACGTGCTC	TGGTCCAAACAGCAATGATTT	NM_000621
human PPIA	GGAGGCTTTGAGGTTTTGCAA	CCTGACATCTAACTGCCAGCA	NM_021130
human RPL13A	TGAAAGCACTCGGAGAATTG	ACAAGATAGGGCCCTCCA	NM_012423
human GAPDH	AGCAAGAGCACAAGAGGAAGAG	TAACTGGTTGAGCACAGGGTAC	NM_002046

Table S2: Antibodies

REAGENT or RESOURCE	SOURCE	IDENTIFIER	DILUITION	
**Primary Antibodies**			**IF**	**WB**
Map2	Synaptic systems	188003	1:200	
Nestin	Thermo Fisher Scientific	A24345	1:100	
Pax6	Thermo Fisher Scientific	A24340	1:100	
SOX1	Thermo Fisher Scientific	A24347	1:100	
SOX2	Thermo Fisher Scientific	A24339	1:100	
OCT4	Thermo Fisher Scientific	A24847	1:100	
SSEA4	Thermo Fisher Scientific	A24866	1:100	
SOX2	Thermo Fisher Scientific	A24759	1:100	
TRA-1-60	Thermo Fisher Scientific	A24868	1:100	
Homer-1	Synaptic systems	160 011	1:100	1:1000
SV2A	Synaptic systems	119 011	1:100	1:1000
PSD95	Synaptic systems	124 011	1:100	1:1000
Synaptophysin 1	Synaptic systems	101 101	1:100	1:1000
TUBB3	Synaptic systems	302 304	1:100	
TUBB3	Sigma	T2200		1:1000
V-GLUT2	Synaptic systems	135 421		1:1000
GAPDH	Santa Cruz	sc:47724		1:1000
GFAP	Millipore	MAB360	1:100	
